# PyPLIF HIPPOS and Receptor Ensemble Docking Increase the Prediction Accuracy of the Structure-Based Virtual Screening Protocol Targeting Acetylcholinesterase

**DOI:** 10.3390/molecules27175661

**Published:** 2022-09-02

**Authors:** Enade P. Istyastono, Florentinus Dika Octa Riswanto, Nunung Yuniarti, Vivitri D. Prasasty, Sudi Mungkasi

**Affiliations:** 1 Faculty of Pharmacy, Sanata Dharma University, Yogyakarta 55282, Indonesia; 2 Department of Pharmacology and Clinical Pharmacy, Faculty of Pharmacy, Universitas Gadjah Mada, Yogyakarta 55281, Indonesia; 3 School of Basic Pharmaceutical and Toxicological Sciences, College of Pharmacy, University of Louisiana Monroe, Monroe, LA 71201, USA; 4 Department of Mathematics, Faculty of Science and Technology, Sanata Dharma University, Yogyakarta 55282, Indonesia

**Keywords:** PyPLIF HIPPOS, AutoDock Vina, receptor ensemble docking, machine learning, drug discovery, acetylcholinesterase

## Abstract

In this article, the upgrading process of the structure-based virtual screening (SBVS) protocol targeting acetylcholinesterase (AChE) previously published in 2017 is presented. The upgraded version of PyPLIF called PyPLIF HIPPOS and the receptor ensemble docking (RED) method using AutoDock Vina were employed to calculate the ensemble protein–ligand interaction fingerprints (ensPLIF) in a retrospective SBVS campaign targeting AChE. A machine learning technique called recursive partitioning and regression trees (RPART) was then used to optimize the prediction accuracy of the protocol by using the ensPLIF values as the descriptors. The best protocol resulting from this research outperformed the previously published SBVS protocol targeting AChE.

## 1. Introduction

The discovery of AChE inhibitors is one of the promising strategies for Alzheimer’s disease (AD) treatment [1,2,3]. The AChE inhibitor donepezil has served as the leading drug in the market since 1996 for dementia treatment in AD patients [4,5,6]. A structure-based structure virtual screening (SBVS) protocol to identify potent inhibitors for acetylcholinesterase (AChE) was introduced in 2017 [7]. The protocol was employed to design chalcone derivatives as AChE inhibitors [7], which were subsequently synthesized and verified in vitro [8]. The protocol was then also employed to screen natural products, which inspired the design of short peptides as AChE inhibitors [9,10]. The prospective screening campaigns of the short peptides [9] have hit peptides AEKY, AERW, AEYQ, AEYT, and AEYTR [11]. The following molecular dynamics simulations and the in vitro test have verified the peptide AEYTR as a potent AChE inhibitor [12]. The snapshots of complex AChE-AEYTR resulting from the molecular dynamics offer the possibility to perform receptor ensemble docking (RED) [13]. On the other hand, several in silico studies involving docking and molecular dynamics to successfully recommend and discover novel AChE inhibitors were recently reported [14,15,16,17].

The flexibility of the AChE was not taken into account in the development of our previously published SBVS protocol [7]. Referring to the lock-and-key theory [18], the SBVS protocol uses a flexible key with particular features to match the rigid lock [7]. There are several methods to include the flexibility of the receptor in SBVS protocols, e.g., the induced-fit docking (IFD) by assigning some residues as flexible [13,19], and the RED by employing selected snapshots from molecular dynamics (MD) simulations [13,20,21]. Employing receptor flexibility could enhance the SBVS protocol [20,21,22].

Software to identify protein–ligand interaction fingerprints (PLIF) called PyPLIF [23] was employed to identify the PLIF, which were derived into ensemble PLIF (ensPLIF) in the development of the SBVS targeting AChE [7]. The descriptor ensPLIF was introduced to circumvent the selection of ligand reference [24] and the possibility of multiple poses of the ligands in the binding pocket [25]. Seven years after the public release of PyPLIF, the upgraded version called PyPLIF HIPPOS was made publicly available in 2020 [26]. PyPLIF HIPPOS was reported 10 times faster compared to its predecessor and had the feature to focus on the interaction with the residue atoms only by neglecting the interaction with the main chain atom of the protein [26]. The research presented in this article aimed to upgrade the SBVS protocol to identify AChE inhibitors [7,10] with better prediction accuracy by taking into account the flexibility of the enzyme. Since PyPLIF HIPPOS was not developed to identify PLIF from docking poses resulting from AutoDock Vina flexible docking [26,27], the RED approach was selected instead of the IFD approach to consider the protein flexibility in this research. The availability of PyPLIF HIPPOS [26] and the AChE-AEYTR complexes resulting from the molecular dynamics simulations [12] were beneficial to attaining the aim.

## 2. Results

The upgrading of the SBVS targeting AChE started with the replication optimization of the retrospective SBVS campaigns. This step reperformed the protocol in the previous SBVS protocol version [7] but used AutoDock Vina [27] instead of PLANTS [28]. The complex for this optimization was the crystal structure 1E66.pdb [29], and the active and decoy compounds for retrospective validation were obtained from the Directory of Useful Decoys, Enhanced (DUD-E) [30]. In the optimization for the upgraded SBVS protocol, the F-measure (F1) values with 1-time, 2-time, 3-time, 4-time, and 5-time replications were 0.088, 0.112, 0.241, 0.241, and 0.237, respectively. Since the 3-time replication provided the optimum predictive activity compared to others, the retrospective SBVS campaign for each clustered AChE-AEYTR was performed three times.

The clustering of the MD snapshots [12] resulted in two AChE-AEYTR complexes for further construction of the SBVS protocol. The complexes were split into the protein (AChE) and the ligand (AEYTR), and then prepared for the docking simulations. The prepared files in the pdbqt format were zipped and are provided here as Supplementary Material S1 (clusters.zip).

**Figure 1:**
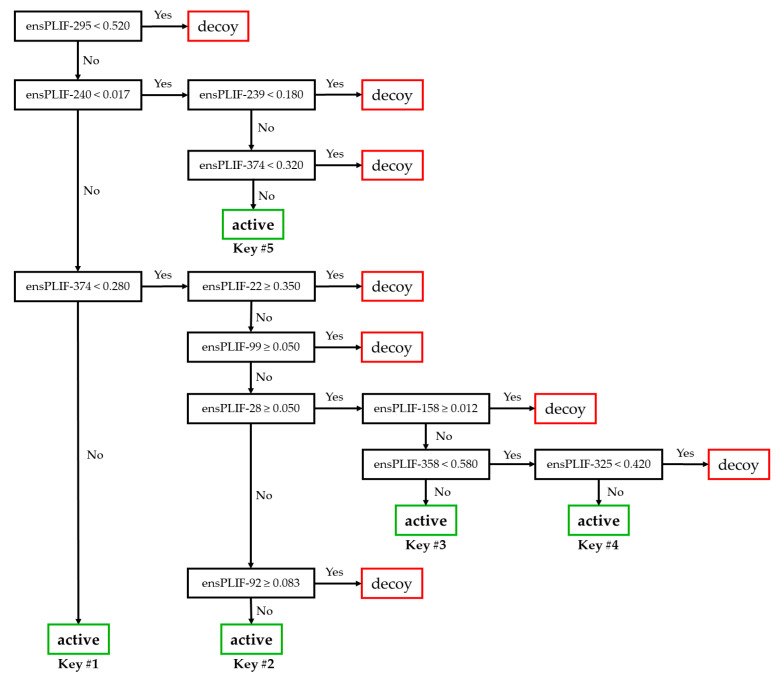
The decision tree resulted from the RPART run with the prior probabilities of 0.9:0.1.

The retrospective SBVS campaigns were performed three times for each cluster. Employing the same docking score-based pose selection as the previously published protocol [7] to optimize the prediction quality of the SBVS protocol reached an F1 value of 0.412 at −8.7 kcal/mol as the maximum cutoff docking score. The F1 value came from the true positive (TP), false negative (FP), true negative (TN), and (false positive) values of 124, 329, 26225, and 25, respectively. The ensPLIF values from the selected poses of the retrospective SBVS campaign with −8.7 kcal/mol as the maximum cutoff docking score are provided in a zipped csv file as Supplementary Material S2 (bestdg.ensplif.nobb.zip). Further optimization of the SBVS protocol by fine-tuning the prior probabilities in running recursive partitioning and regression trees (RPART) on Supplementary Material S2 identified that prior probabilities of 0.9:0.1 resulted in the highest balanced accuracy (BA) value (0.729) among other runs with the F1 value of more than 0.413. The optimized protocol had the enrichment factor (EF), F1, and BA values of 34.068, 0.415, and 0.729, respectively. These values came from the TP, FN, TN, and FP values of 214, 239, 25886, and 364, respectively. Overfitting, cross-correlation, and chance correlation were not observed in the decision tree model (Figure 1) resulting from the optimized RPART run.

Based on Figure 1 and Table 1, there are five branches leading to the identification of active compounds as AChE inhibitors. These branches reflect five “keys” to open the AChE “lock”. There are 11 ensPLIF descriptors that play an important role, i.e., ensPLIF-22, -28, -92, -99, -158, -239, -240, -295, -325, -358, and -374. In AChE, these ensPLIF descriptors related to the hydrophobic interaction to Asp72, the ionic interaction with Asp72 as the anion, the hydrophobic interaction to Asn85, the hydrophobic interaction to Pro86, the H-bond with Ser112 as the donor, the hydrophobic interaction to Trp279, the aromatic face-to-face interaction to Trp279, the hydrophobic interaction to Phe330, the aromatic edge-to-face interaction to Tyr334, the hydrophobic interaction to His440, and the aromatic edge-to-face interaction to Tyr442, respectively (Table 1). The decision tree (Figure 1) indicates that the hydrophobic interaction to Asp72, the ionic interaction with Asp72 as the anion, the hydrophobic interaction to Asn85, the hydrophobic interaction to Pro86, and the H-bond with Ser112 as the donor, are unfavorable interactions. Both the previous version [7] and the version developed here have 11 ensPLIF descriptors. The following are the descriptors from the previous version [7]: ensPLIF-29, -193, -204, -208, -297, -302, -316, -337, -358, -365, and -386, which are related to the hydrophobic interaction to Gln74, the H-bond to Tyr130 (protein as the donor), the hydrophobic interaction to Ser200, the H-bond to Ser200 (protein as the acceptor), the aromatic edge-to-face interaction to Phe330, the hydrophobic interaction to Phe331, the hydrophobic interaction to Leu333, the hydrophobic interaction to Trp432, the hydrophobic interaction to His440, the hydrophobic interaction to Gly441, and the hydrophobic interaction to Ile444, respectively.

**Figure 2:**
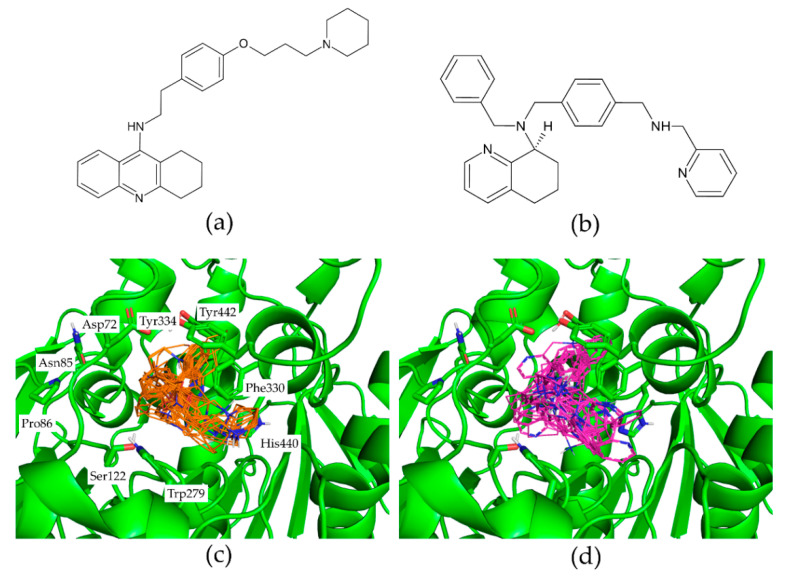
The representative of the active compound ChEMBL15056 (**a**) and the decoy compound C49071124 (**b**). The representative docking results of ChEMBL15056 (carbon atoms are in orange) and C49071124 (carbon atoms are in magenta) in the AChE binding pocket are presented in (**c**) and (**d**), respectively. Figures (**c**,**d**) were prepared by employing PyMOL version 2.5.2 (https://pymol.org/2/; accessed on 23 May 2022).

Figure 2 presents ChEMBL15056 and C49071124 as the representative of active and decoy compounds, respectively. Based on the docking poses, the ensPLIF-22, -28, -92, -99, -158, -239, -240, -295, -325, -358, and -374 values of ChEMBL15056 were 0.467, 0.033, 0, 0, 0.3, 0.4, 0.033, 0.933, 0.433, 0.3, and 0.533. These ensPLIF values indicate that ChEMBL15056 is one of the Key #1 to open the AChE lock (Figure 1). On the other hand, the ensPLIF-22, -28, -92, -99, -158, -239, -240, -295, -325, -358, and -374 values of C49071124 are 0.267, 0.1, 0, 0, 0.067, 0.1, 0.1, 0.767, 0.4, 0.567, and 0.133, respectively. These ensPLIF values do not correspond to any key to open the AChE lock (Figure 1).

## 3. Discussion

The SBVS protocol targeting AChE released in 2017 [7] played an important role in the discovery of the pentapeptide AEYTR as a potent AChE inhibitor with IC50 value of 0.462 ± 0.079 nM [9,10,11,12]. The backbone of the protocol is PLANTS docking software [28] and PyPLIF [23]. In the previous SBVS protocol [7], every compound is docked five times independently, resulting in 250 poses to derive ensPLIF descriptor for the corresponding compound [7]. Since the RED approach uses more than 1 receptor coordinate [20,21], the replication should be optimized to avoid redundancy in the SBVS protocol, which in turn optimizes the computational cost of the SBVS protocol. The optimization employing the same protocol with [7] but using AutoDock Vina [27] and PyPLIF HIPPOS instead of PLANTS and PyPLIF, respectively, concluded that the replication of three times is the optimum one.

The discovery process of AEYTR as a potent AChE inhibitor involved MD simulations [11,12], which provided snapshots of AChE-AEYTR complex. The clustering of the production runs snapshots performed in this research resulted in 2 different AChE-AEYTR complexes, which indicated the stability of the AChE-AEYTR complex. This is in line with the results presented previously [11,12]. Together with the optimized 3-times of the docking replication, these 2 AChE-AEYTR complexes require in total 6-times independent runs for each screened compound.

The residues Phe330 and His440 are suggested as the molecular determinant of the AChE inhibitor binding since both residues play an important role in the previous [7] and the upgraded protocol. The importance of the residue Phe330 in the AChE inhibitor binding was recently reported also by Liu et al. [15], Daoud et al. [14], and van der Westhuizen [16], while the importance of the residue His440 was reported by van der Westhuizen [16]. This reflects the hydrophobic and aromatic nature of the ligand reference in the complex used in the SBVS development. The previous version uses a crystal structure 1E66.pdb with huprine X (HUX) as the co-crystal ligand [7,29,30], while this upgraded version uses AChE-AEYTR resulted from the MD simulations [12]. Both HUX and AEYTR have aromatic moiety in their structures. Notably, for Phe330, the interaction type in the decision tree in the previous version is the aromatic (edge-to-face) [7], but in the upgraded version is hydrophobic. All hits in the previous prospective screening on small peptides [9] have aromatic moiety in their structure [11]. Moreover, the marketed AChE inhibitor donepezil has two aromatic moieties in its structure [5,12]. Therefore, it is highly suggested in the prospective screening campaign targeting AChE or in the hit-to-lead optimization to focus on compounds with aromatic moiety.

Based on Table 2, the upgraded version of the SBVS protocol developed here has the F1 value of 0.415, which is slightly better compared to the previous version (0.413). Nevertheless, the upgraded version outperforms the previous version in terms of the BA and the sensitivity values. The BA values of the previous and the upgraded version are 0.636 [7] and 0.729, respectively, while the sensitivity values are 0.274 [7] and 0.472, respectively. During the preparation of the manuscript, SBVS protocols targeting AChE, which were retrospectively validated using the DUD-E dataset, were published and successfully identify novel inhibitors [16]. The EF, F1, and BA values of the upgraded SBVS protocol developed in this article are better compared to those values of the best SBVS protocol in van der Westhuizen et al. [16].

The upgraded version presented in this article would be employed further for prospective screening in the discovery of AChE inhibitors. Notably, the DUD-E dataset has been recently optimized into the newest version called DUDE-Z [31], which offers an opportunity to benchmark the upgraded version of the SBVS targeting AChE presented here. Moreover, the ensPLIF descriptor resulting from the combination of PyPLIF HIPPOS and RED approach has become a routine in our ongoing research project on the discovery of novel dipeptidyl peptidase IV (DPP4) inhibitors.

## 4. Materials and Methods

### 4.1. Materials

The AChE crystal structure with HUX as the co-crystalized ligand 1E66.pdb obtained from https://www.rcsb.org/structure/1e66 (accessed on 31 March 2021) [29] was used in the optimization of the docking replication number. The AchE-AEYTR complexes resulted from the MD simulations snapshots were obtained from [12]. The active set of AchE inhibitors (actives_final.ism) and the decoy set (decoys_final.ism) in the SMILES form were downloaded from http://dude.docking.org/targets/aces (accessed on 31 March 2021) [30]. The computational simulations were performed in a 64-bit Linux (CentOS 7) server with 16 cores of Intel® Xeon® CPU E5-2620 v4 @ 2.10GHz and 16 GB of RAM. The main software in this machine involved in this research was YASARA-Structure version 21.12.19 [32], AutoDock Vina version 1.1.2 [27], PyPLIF HIPPOS version 0.1.2 (https://github.com/radifar/PyPLIF-HIPPOS/releases/tag/0.1.2, accessed on 20 March 2021) [33], PLANTS docking software 1.2 [28,34], SPORES 1.3 [35], ADFRsuite 1.0 [19], and RPART package [36] in R statistical computing software version 3.6.0 [37].

### 4.2. Methods

#### 4.2.1. Ligand Preparation

The files actives_final.ism (453 compounds) and decoys_final.ism (26250 compounds) were downloaded from http://dude.docking.org/targets/aces (accessed on 31 March 2021) [30]. The second column of the file actives_final.ism was removed. The rest of SMILES structure lines from actives_final.ism were appended to the file decoys_final.ism, and then the file was stored as dude_aces.smi. A macro file in the same directory as the file dude_aces.smi was created to convert the structures from SMILES to pdb. The following is the summary of the macro file: “The simulation was run with YASARA. The SMILES for each line in dude_aces.smi was built into its three-dimensional (3D) form. The pH system was set to pH 7.4, and the hydrogens were updated. The compound was then energy minimized using NOVA as the FF [38]. The optimized structure was saved as a pdb file.” The resulting pdb files were then subjected to the module “prepare_ligand” from ADFRsuite to be converted into the in pdbqt files readily for molecular docking simulations using AutoDock Vina [27]. During this pdb to pdbqt conversion step using ADFRsuite, 20 pdb structures of the decoys could not be converted into their pdbqt format. Since these compounds would not result in any docking poses, they were then predicted as inactive (N) in the further analysis.

#### 4.2.2. Replication Optimization

The protein preparation and the predictive ability calculation for this replication optimization followed the method reported in [7]. The modification of the method was in the ligand preparation process, the molecular docking software, and the PyPLIF version used in this step. The ligands here were prepared as presented previously (Section 4.2.1), the molecular docking software used AutoDock Vina instead of PLANTS, while the PyPLIF version was PyPLIF HIPPOS instead of PyPLIF.

#### 4.2.3. Proteins Preparation

The AchE-AEYTR complexes resulted from the MD simulations snapshots obtained from [12] in pdb formats were clustered using YASARA-Structure with the minimum heavy-atom RMSD between different clusters of 2.0 Å. Each cluster was energy minimized and split into a pdb file for the AchE part and a mol2 file for AEYTR part. These files were converted to pdbqt using ADFRsuite and provided here as Supplementary Material S1.

#### 4.2.4. Automated Molecular Docking Simulations Using AutoDock Vina

The configuration for the docking simulations was set as follows: num_modes = 5, energy_range = 5, and cpu = 4, while the other options were left default. The pdb files from Section 4.2.3 were converted to mol2 files using “complete” and followed by “reprot” modules from SPORES [35]. Together with the mol2 files from Section 4.2.3, these mol2 files were used to define the binding pocket of each cluster. The center of the AEYTR of each virtual target was set as the XYZ coordinate position, and the distance of 5 Å from the surface of the residue was used to calculate the docking box size. The module “bind” from PLANTS was used to obtain the values of the XYZ coordinate positions and the size of the docking boxes. All prepared ligands were docked three times to all clusters using AutoDock Vina [27] in parallel made use of the 16 processors from the server.

#### 4.2.5. Ensemble Docking Scores and ensplif Calculations

The ensemble docking scores and ensPLIF calculations were performed in the server using a similar method published by Istyastono et al. [33]. The configuration file to perform PLIF identification by PyPLIF HIPPOS required a list of residues in the binding pocket [26]. Since the configuration file used for the docking results from different clusters, a consensus list of residues was created by combining all unique residues identified by using the module “bind” from PLANTS (see Section 4.2.4). The following was the consensus list of residues used in the configuration file to run PyPLIF HIPPOS: Gln69, Tyr70, Val71, Asp72, Gln74, Phe75, Phe78, Ser79, Gly80, Ser81, Glu82, Met83, Trp84, Asn85, Pro86, Trp114, Tyr116, Gly117, Gly118, Gly119, Phe120, Tyr121, Ser122, Gly123, Ser124, Leu127, Val129, Tyr130, Glu199, Ser200, Ala201, Gly202, Ser226, Trp233, Trp279, Phe288, Phe290, Asn324, Asp326, Glu327, Gly328, Ser329, Phe330, Phe331, Leu332, Leu333, Tyr334, Val400, Trp432, Met436, Ile439, His440, Gly441, Tyr442, Glu443, Ile444, and Glu445.

By employing the configuration files, the PLIF identifications were performed for all docking poses resulted from the retrospective SBVS (see Section 4.2.4). The option “nobb” to neglect the interaction with the backbone atoms of the protein was used [26]. Subsequently, employing the similar procedure presented by Istyastono et al. [33], ensPLIF values were calculated. The results were then arranged in a table for each receptor to be easily analyzed using the RPART package in R (Supplementary Material S2). The tables started with the first column named “y” encoding the observed data (“1” for active; “0” for decoy), followed by “name” for the name of the corresponding ligand, “dg” for the average docking scores (kcal/mol) resulted from the docking simulations (see Section 4.2.4), and then ensPLIF variables (“V1” for ensPLIF-1, “V2” for ensPLIF-2, until the whole ensPLIF values were covered).

#### 4.2.6. Analysis Using RPART in R

The analysis to provide the best decision tree with the highest BA value was performed using R version 3.6.0 in the server. The prior probabilities were optimized in this analysis. The best decision tree resulting from the RPART analysis was then examined for possibilities of overfitting [39], the cross-correlation between identified ensPLIF variables [40], and chance correlation [33].

## 5. Conclusions

The upgraded version of the SBVS protocol targeting AChE using the PyPLIF HIPPOS and RED approaches outperforms the previously published one in 2017. The upgraded version should be used as a substitute for the previous one in performing prospective screening. Compounds with aromatic moiety are suggested to be the focus of the prospective screening using the upgraded SBVS protocol.

## Figures and Tables

**Table 1:** The ensPLIF descriptors in the decision tree resulted from the RPART run with the prior probabilities of 0.9:0.1.

Variable No.	Descriptor	Corresponding Residue	Corresponding Interaction Type ^1^
V22	ensPLIF-22	Asp72	hydrophobic
V28	ensPLIF-28	Asp72	ionic (residue as the anion)
V92	ensPLIF-92	Asn85	hydrophobic
V99	ensPLIF-99	Pro86	hydrophobic
V158	ensPLIF-158	Ser122	H-bond (residue as the donor)
V239	ensPLIF-239	Trp279	hydrophobic
V240	ensPLIF-240	Trp279	aromatic (face-to-face)
V295	ensPLIF-295	Phe330	hydrophobic
V325	ensPLIF-325	Tyr334	aromatic (edge-to-face)
V358	ensPLIF-358	His440	hydrophobic
V374	ensPLIF-374	Tyr442	aromatic (edge-to-face)

^1^ Refers to [23,26].

**Table 2:** Prediction abilities of some SBVS protocols to identify AChE inhibitors using ligand and decoys from DUD-E [30].

SBVS Protocol	Confusion Matrix				Statistical Significance		
	TP	FN	TN	FP	EF	F1	BA
Mysinger et al. [30] ^1^	91	362	25988	262	20.1	0.225	0.595
Riswanto et al. [7]	124	329	26226	24	299.393	0.413	0.636
van der Westhuizen et al. [16] ^1^	127	326	25988	262	28	0.301	0.635
SBVS developed in this article	214	239	25886	364	34.068	0.415	0.729

^1^ Calculated from the best EF_1%_ values reported in the article.

## Data Availability

The data presented in this study are available in this article as Supplementary Materials.

## Supplementary Materials

The following supporting information can be downloaded at: https://www.mdpi.com/article/10.3390/molecules27175661/s1, Supplementary Material S1: clusters.zip; Supplementary Material S2: bestdg.ensplif.nobb.zip.
